# Genetic Polymorphisms Influencing Arsenic Metabolism: Evidence from Argentina

**DOI:** 10.1289/ehp.9734

**Published:** 2007-01-08

**Authors:** Karin Schläwicke Engström, Karin Broberg, Gabriela Concha, Barbro Nermell, Margareta Warholm, Marie Vahter

**Affiliations:** 1 Division of Occupational and Environmental Medicine, Department of Laboratory Medicine, Lund University, Lund, Sweden; 2 Toxicology Division, National Food Administration, Uppsala, Sweden; 3 Section for Metals and Health and; 4 Division of Work Environment Toxicology, Institute of Environmental Medicine, Karolinska Institutet, Stockholm, Sweden

**Keywords:** arsenic, *AS3MT*, *GSTM1*, *GSTO1*, *GSTT1*, metabolism, methylation, *MTHFR*, *MTR*, polymorphisms

## Abstract

The susceptibility to arsenic-induced diseases differs greatly between individuals, possibly due to interindividual variations in As metabolism that affect retention and distribution of toxic metabolites. To elucidate the role of genetic factors in As metabolism, we studied how polymorphisms in six genes affected the urinary metabolite pattern in a group of indigenous women (*n* = 147) in northern Argentina who were exposed to approximately 200 μg/L As in drinking water. These women had low urinary percentages of monomethylated As (MMA) and high percentages of dimethylated As (DMA). MMA has been associated with adverse health effects, and DMA has the lowest body retention of the metabolites. The genes studied were arsenic(+III)methyltransferase (*AS3MT*)*,* glutathione *S*-transferase omega 1 (*GSTO1*), 5-methyltetrahydrofolate-homocysteine methyltransferase (*MTR*), methylenetetrahydrofolate reductase (*MTHFR*)*,* and glutathione *S*-transferases mu 1 (*GSTM1*) and theta 1 (*GSTT1*). We found three intronic polymorphisms in *AS3MT* (G12390C, C14215T, and G35991A) associated with a lower percentage of MMA (%MMA) and a higher percentage of DMA (%DMA) in urine. The variant homozygotes showed approximately half the %MMA compared with wild-type homozygotes. These polymorphisms were in strong linkage, with high allelic frequencies (72–76%) compared with other populations. We also saw minor effects of other polymorphisms in the multivariate regression analysis with effect modification for the deletion genotypes for *GSTM1* (affecting %MMA) and *GSTT1* (affecting %MMA and %DMA). For pregnant women, effect modification was seen for the folate-metabolizing genes *MTR* and *MTHFR*. In conclusion, these findings indicate that polymorphisms in *AS3MT*—and possibly *GSTM1*, *GSTT1*, *MTR*, and *MTHFR—*are responsible for a large part of the interindividual variation in As metabolism and susceptibility.

Millions of people around the world drink water with elevated concentrations of arsenic, a potent human carcinogen. It is well established that chronic exposure to As is associated with skin, lung, and bladder cancers [International Agency for Research on Cancer (IARC) 2004; National Research Council (NRC) 2001] as well as vascular diseases, hepatotoxicity, diabetes, and chronic cough (Mazumder et al. 2005; NRC 2001).

Inorganic As (iAs) is metabolized by reduction of pentavalent iAs to trivalent, followed by oxidative methylation to monomethylated As (MMA), further reduction from pentavalent MMA to trivalent, and at last methylation to dimethylated As (DMA) (Vahter 2002). Not all of the As is fully methylated and some As remains as iAs and MMA, which are eliminated in the urine along with DMA. *In vitro* studies have shown that the trivalent forms, particularly monomethylarsonous acid (MMA^III^), are generally more toxic than the pentavalent forms (Nesnow et al. 2002; Styblo et al. 2000; Vega et al. 2001). Thus, the observed large variability in the distribution of metabolites in urine among individuals and population groups might be associated with differences in tissue distribution and retention of toxic metabolites (Vahter 2002). This, in turn, is likely to lead to variation in susceptibility. A number of studies indicate that increased percentage of MMA (%MMA) and decreased percentage of DMA (%DMA) in urine are associated with an increase in As retention in the body (Vahter 2002) and an increase of toxic effects of As (Chen et al. 2003a, 2003b; Del Razo et al. 1997; Mäki-Paakkanen et al. 1998; Steinmaus et al. 2006; Tseng et al. 2005; Yu et al. 2000).

Part of the interindividual variation in As metabolism may be due to environmental factors, but earlier studies have shown that hereditary differences are very likely to contribute (Chung et al. 2002; Vahter 2000). So far, knowledge on these genetic factors and their mechanisms is very limited. Recent findings from small-scale studies indicate that polymorphisms in glutathione *S*-transferase (GST) omega 1 (*GSTO1*), which encodes an enzyme that can reduce pentavalent As species, might be related to enzyme activity and patterns of methylated As metabolites (Marnell et al. 2003; Tanaka-Kagawa et al. 2003). A few studies have indicated that two other GSTs, GST mu 1 (GSTM1) and GST theta 1 (GSTT1), have minor effects on As metabolism as well (Chiou et al. 1997; Marcos et al. 2006). In another study, involving a Mexican population, arsenic(+III)methyl-transferase (*AS3MT*; also labeled *CYT19*), which is known to methylate trivalent As, was found to have three intronic single nucleotide polymorphisms (SNPs) that were significantly related to DMA/MMA ratios. However, this association was only valid for children (Meza et al. 2005). *S*-Adenosyl-methionine (SAM) is the methyl donor for methylation of As (Marafante et al. 1985), and polymorphisms in genes involved in SAM metabolism (i.e., one-carbon metabolism), for example, methylene-tetrahydrofolate reductase (*MTHFR*) and 5-methyltetrahydrofolate-homocysteine methyltransferase (*MTR*), may affect As metabolism.

Our aim was to elucidate how variations in the various As metabolism-related genes affect the distribution of As metabolites. Therefore, we genotyped and phenotyped inhabitants from a city in the northern Argentinean Andes, where the inhabitants are exposed to high levels of As and have an unusual metabolism (Vahter et al. 1995). The genes of interest were selected by *a*) searching for new and previously reported polymorphisms in *GSTO1* and *AS3MT* by sequencing; and *b*) screening for previously reported polymorphisms in *GSTM1, GSTT1, GSTO1, MTR*, and *MTHFR*.

## Materials and Methods

### Study areas and populations

Participants in this study were women living in San Antonio de los Cobres (SAC), a village in the northern Argentinean Andes. Men were excluded from the study because they often worked in other geographic locations for extended periods of time. All individuals in SAC used drinking water from the same source; the As concentration of this water was around 200 μg/L (with little variation over time) because of the As content of the bedrock (Concha et al. 2006). Urine and blood samples were collected from a total of 148 women in 1997, 2004, and 2005. The metabolite data from 1997, but no genotype data, were published previously (Concha et al. 1998b). The group studied in 1997 included pregnant women only. For analysis of associations between genotype and As metabolism, we pooled all of the data for women studied in 2004 and 2005 into one group (*n* = 111). Metabolite data was missing for one individual. Because the metabolite pattern changes during pregnancy (Concha et al. 1998b), all pregnant women (from 1997 from 2004, and 2005) formed a separate group (*n* = 37).

For women sampled in 2004–2005, data were available on age (mean, 35; range, 15–76 years), weight (mean, 58; range, 40–88 kg), body mass index (BMI; mean, 25; range, 17–38 kg/m^2^), and coca usage (49% were users). For pregnant women, data were available on age (mean, 25; range, 16–39 years) and gestational week (GW; mean, 25; range, 10–37 weeks). The subjects were recruited via the local radio announcements and hospital registers and were interviewed about history of residence, residences of parents and grandparents, parity, and water consumption. The interviews revealed that individuals from SAC were mainly of indigenous (Atacameño) origin, but there was a variation in Hispanic and indigenous origin. All individuals were genetically unrelated.

The Health Ministry of Salta, Argentina, and the Ethics Committee of the Karolinska Institutet approved this study. Before sampling, the responsible community health worker and the interviewer (one of the authors) informed the women of all details of the research project, and all study participants gave written informed consent.

### Arsenic analysis

We determined values of As in water and total concentration of As metabolites in urine (U-As) using hydride generation-atomic absorption spectrophotometry (HG-AAS) (Vahter et al. 1986). Details of sample collection have been reported previously (Concha et al. 1998a). The urine samples were frozen at the hospital in SAC and kept frozen (−20°C) for up to 5 months (the 2005 samples were frozen less than 1 month) until analysis. The speciation of As metabolites (iAs, MMA, and DMA) in urine for assessment of the metabolite pattern was performed using ion-exchange chromatography as described by Vahter et al. (1995). For the samples collected in 2004 and 2005, the As metabolites were speciated using HPLC-HG-inductively coupled plasma mass spectroscopy (ICPMS) (Agilent 1100 series system and Agilent 7500ce; Agilent Technologies, Waldbronn, Germany) (Lindberg et al. 2006). Quality control for the HG-AAS method has been reported previously (Concha 2001; Crecelius and Yager 1997; Vahter et al. 1995). In 2002, we participated in two interlaboratory comparisons within the European Union Arsenic Health Risk and Molecular Epidemiology (ASHRAM) project, organized by W. Goessler (University of Graz, Graz, Austria), with good results (Vahter et al. 2006). After the introduction of the HPLC-HG-ICPMS method, we performed intralaboratory comparisons using HG-AAS, HPLC-HG-atomic fluorescence spectrometry, and HPLC-HG-ICPMS, showing excellent agreement (Lindberg et al. 2007; Vahter et al. 2006). We used HPLC-HG-ICPMS to reanalyze nine remaining urine samples collected in SAC in 1994 (Concha et al. 1998a). The correlation between these new results compared with the 10-year-old results was 0.97 (%MMA, 0.7–8.3%; concentration range, 2–37 μg As/L as MMA). To compensate for variations in dilution of urine, the As concentrations were adjusted to the mean specific gravity of 1.019 g/mL, measured by a hand refractometer.

### Polymorphism detection and analysis

DNA was isolated from either whole blood by a modified salting-out method (Miller et al. 1988), or from buccal cells (by buccal swabs) using a QIAamp DNA Mini Kit (Qiagen, Hilden, Germany); this extraction was performed at the Swegene DNA Extraction Facility at Malmö University Hospital (Malmö, Sweden).

### Initial screening

For *GSTO1* [UniGene Accession no. Hs.190028; National Center for Biotechnology Information (NCBI) 2006c] and *AS3MT* (UniGene, Hs.123461)*,* sequencing was performed for all exons (apart from exon 2 of *AS3MT*) and flanking regions for 37 selected subjects with low %MMA, from both groups (2004–2005 group and pregnant group). Polymorphisms with a variant allele frequency > 20% were thereafter analyzed in the entire population. For sequence analysis, we followed the standard protocol of Applied Biosystems (Protocol no. 4339920A). We performed polymerase chain reaction (PCR) cycle-sequencing reactions using Big Dye Terminator, version 3.1, Cycle-Sequencing Premix (Applied Biosystems); the DyeEx 96 kit (Qiagen, Hilden, Germany) was used for removal of unincorporated dye terminators. For the other genes studied, all individuals were genotyped by different methods. Primers for *GSTO1* were chosen according to Marnell et al. (2003) with some modifications, based on the sequence of accession no. AY817669 (NCBI 2006a). We chose primers for *AS3MT* based on the sequence AY817668. SNPs in *GSTO1* and *AS3MT* refer to positions within these sequences. All primers, annealing temperatures, PCR and sequencing conditions, and quality control information are available upon request. The sequencing products were separated with an ABI 3730 DNA Analyzer (Applied Biosystems) at the Swegene Resource Center for Profiling Polygenic Diseases (Malmö University Hospital). Obtained sequence chromatograms were analyzed with ChromasPro (Technelysium Pty Ltd., Tewantin, Australia) and compared with previously described polymorphic sites using the SNP Database (NCBI 2006b). For samples with variations from the consensus sequence, we performed a new PCR of genomic DNA followed by a sequencing reaction from the opposite strand.

### Genotyping of all samples

We performed sequencing for SNPs 12390 and 14215 in *AS3MT*. The sequencing fragment for 14215 also included Met287Thr, which had an allele frequency < 20% in the initial screening. We analyzed G35991A by allelic discrimination using the Taqman assay (Applied Biosystems). The deletion alleles for *GSTM1* (UniGene, Hs.301961) and *GSTT1* (UniGene, Hs.568022), as well as GSTM1*0 and GSTT1*0 (homozygotes for the deletion), were analyzed by multiplex PCR reaction (Hou et al. 1995; Pemble et al. 1994). Restriction fragment length polymorphism (RFLP) analysis was performed for Asp919Gly in *MTR* (UniGene, Hs.498187) (Leclerc et al. 1996) and Ala222Val in *MTHFR* (UniGene, Hs.214142) (Frosst et al. 1995). Furthermore, samples taken year 2004 were genotyped for Glu155del (deletion of glutamate 155) and Thr217Asn in *GSTO1* with RFLP. Linkage disequilibrium (LD) calculations were performed using Haploview 3.32 (Barrett et al. 2005).

### Statistical analyses

We tested deviation from Hardy-Weinberg equilibrium using chi-square analysis. Associations between genotypes (independent variables) and urinary As metabolites (dependent variables) were analyzed with one-way analysis of variance (ANOVA). For subsequent post hoc tests, adjustments for multiple testing between subgroups (i.e. genotypes) were made with Bonferroni correction. The values for metabolites used in the statistical analyses were %iAs, %MMA, %DMA, MMA/iAs ratio (first methylation step), DMA/MMA ratio (second methylation step), and U-As [all natural log (ln) transformed]. *GSTT1* and *GSTM1* genotypes were divided into two groups: homozygotes for the deletion, GSTM1*0/GSTT1*0; or those carrying at least one allele, GSTM1*1/GSTT1*1. Furthermore, multivariate regression analyses were performed with the same dependent variables as in the ANOVA analysis except for U-As, which in this case was used as an independent variable (exposure marker). For each dependent variable, we tested each potentially influential independent variable [U-As, weight, BMI, age, coca usage (yes/no), and GW (pregnant women only)]; an independent variable was included that provided a *p*-value < 0.2. The final models were performed with an interaction term between the main independent variable and genotype. Data from individuals with no variant alleles (0) were used as references values and data from individuals with one or two variant alleles were compared with these values. For *GSTM1* and *GSTT1*, we used individuals with GSTM1*1/GSTT1*1 as referents. All statistical analyses were performed using SPSS (version 13; SPSS, Chicago, IL, USA).

## Results

### Genotypic background

For *AS3MT*, all exons except exon 2 were successfully sequenced. We found nine previously reported polymorphisms (bNCBI SNP Database 2006b) in the initial screening. Out of these, three had a frequency > 20% (Table 1): G12390C (a G-to-C substitution at nucleotide position 12390, intron 5), C14215T (intron 7), and G35991A (intron 9). These variant alleles were in significant LD (*r*^2^ = 0.95 for positions 12390 and 14215; 0.74 for positions 12390 and 35991; and 0.78 for positions 14215 and 35991). Of the remaining six polymorphisms, one was an exonic polymorphism (Met287Thr), and five were intronic [C5019T, a 36-bp deletion at 5051, T5194G, a deletion of TTT at 8788, and T12590C].

For *GSTO1*, we found no polymorphisms with an allele frequency > 20%. However, we did identify a novel genetic variant, T2562C (one heterozygous carrier), and we verified five previously reported polymorphisms—three exonic (Ala140Asp, Glu155del, and Glu208Lys), and two intronic (A2207G and a GGC deletion at 2609). These *GSTO1* polymorphisms were present in one to three individuals, mostly in heterozygous form, with allele frequencies between 1.2–6.0%. The frequencies of polymorphisms in other genes studied are shown in Table 1 together with average allelic frequencies among other populations studied with similar ancestry (i.e., indigenous or European ancestry).

### Metabolite pattern and genotype

The urinary metabolite patterns for the group of 2004–2005 women and for pregnant women are shown in Table 2. We did not find trivalent MMA^III^ in any of the urine samples, and we did not look for dimethylarsinous acid (DMA^III^), because any small amounts present would have been oxidized before freezing.

**Figure 1:**
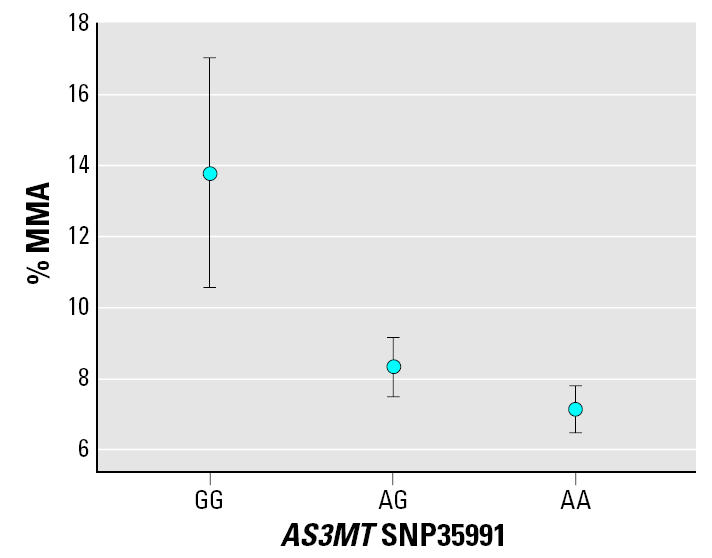
%MMA for *AS3MT* G35991A genotypes. Error bars indicate 95% confidence intervals.

When performing ANOVA, we found a statistically significant association between the genotypes *AS3MT* G12390C, C14215T, and A35991G and the As metabolite pattern for the 2004–2005 group (A35991G is shown in Figure 1). The variant alleles were associated with a decrease in %MMA and an increase in %DMA in urine, and consequently a higher ratio for the second methylation step. This was valid for each of these SNPs separately (Table 3, data shown only for SNP35991 because the results for all three SNPs were very similar, with *p*-values < 0.001 for %MMA, %DMA, and DMA/MMA ratio for all SNPs). We found a difference between all genotypes: the variant homozygotes displayed the lowest %MMA and the highest %DMA. The effect was strongest for %MMA, where the difference was statistically significant between all genotypes for SNP12390, SNP14215, and SNP35991, except for the comparison between heterozygotes and variant homozygotes for SNP35991 (*p* = 0.10). The %MMA for SNP35991 is shown in Table 1, and this SNP is presented in Table 3 (ANOVA). The %iAs showed the same pattern as %MMA, but this result was not statistically significant, and neither was the MMA/iAs ratio (first methylation step).

The pattern was also valid for all *AS3MT* SNPs for pregnant women, where the difference in %DMA between SNP35991 wild types and variant homozygotes was statistically significant (Table 4), whereas the remaining analyses did not reach statistical significance (data not shown). We found no difference in U-As concentration between the different genotypes. We analyzed arsenic metabolite pattern and variant allele frequencies in a subset of individuals with a documented indigenous origin (based on interviews) in the 2004–2005 group (*n* = 76). In this group, the allelic frequency for the variant allele of 35991 was 77.7%. The differences in %MMA between the genotypes were even higher (e.g., for 35991, where the mean %MMA for GG was 15.5% and for AA 5.7% compared to 13.6% and 6.3%, respectively, for the entire 2004–2005 group). For the other genes studied, no such differences were seen. Among the individuals with less documented indigenous origin (*n* = 34) the variant allelic frequency was 72% and the %MMA was 11.5% for GG and 8.7% for AA.

Among the 2004–2005 women, variant homozygotes of *MTR* had lower %DMA and higher %MMA, and thus lower DMA/MMA ratios (Table 3). However, there were only two variant homozygotes. Pregnant women carrying one variant allele of *MTR* had lower %DMA (Table 4) and higher %iAs, although the differences were not statistically significant. The pregnant women also displayed lower %DMA (*p* = 0.051) for GSTT1*0.

**Figure 2:**
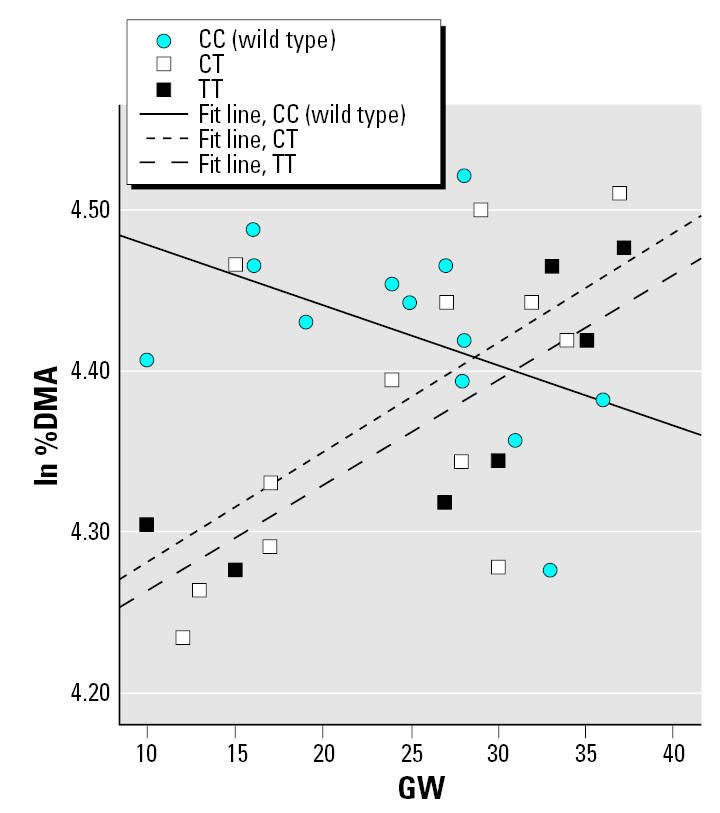
%DMA (ln transformed) as a function of GW among pregnant women with different *MTHFR* Ala222Val genotypes. Simple regression lines within each genotype group are presented (the regression lines were not derived from the multivariate regression model presented in Table 5; rather, they illustrate the general direction of interaction).

**Figure 3:**
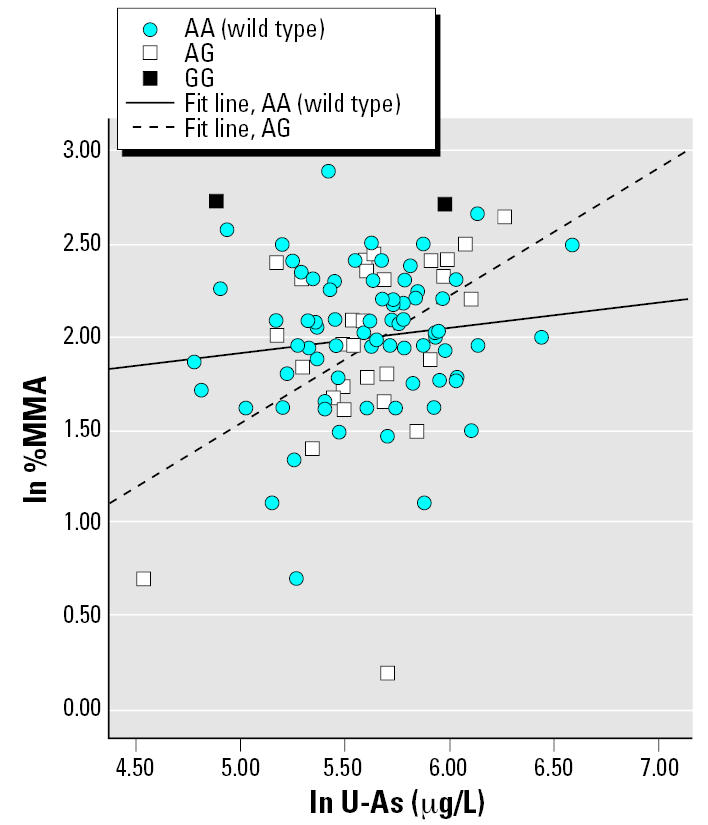
%MMA (ln transformed) as a function of U-As (μg/L) among 2004–2005 women with different *MTR* Ala919Gly genotypes. Simple regression lines within each genotype group are presented (the regression lines were not derived from the multivariate regression model presented in Table 5; rather, they illustrate the general direction of interaction).

**Figure 4:**
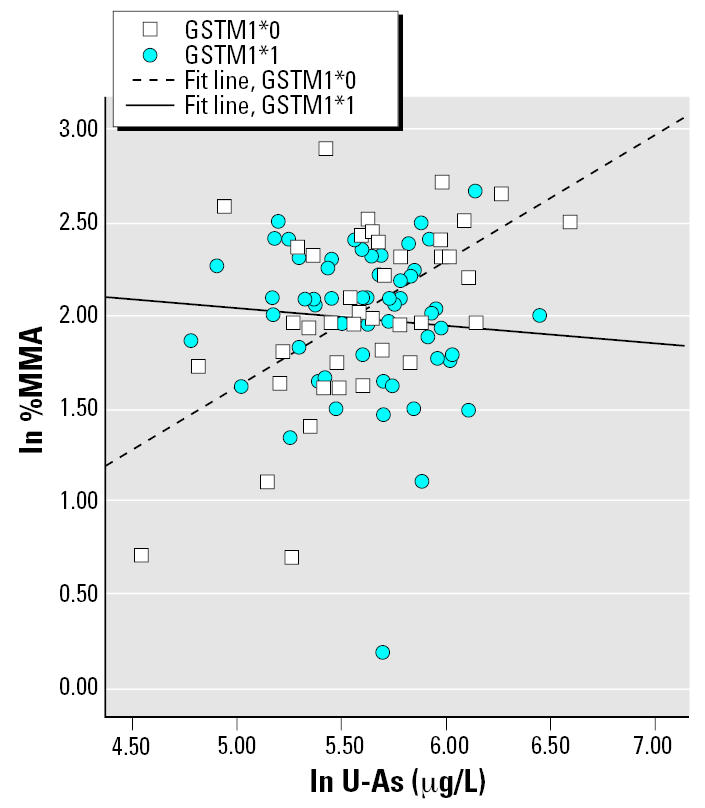
%MMA (ln transformed) as a function of U-As (μg/L) among 2004–2005 women with different *GSTM1* genotypes. Simple regression lines within each genotype group are presented (the regression lines were not derived from the multivariate regression model presented in Table 5; rather, they illustrate the general direction of interaction).

**Figure 5:**
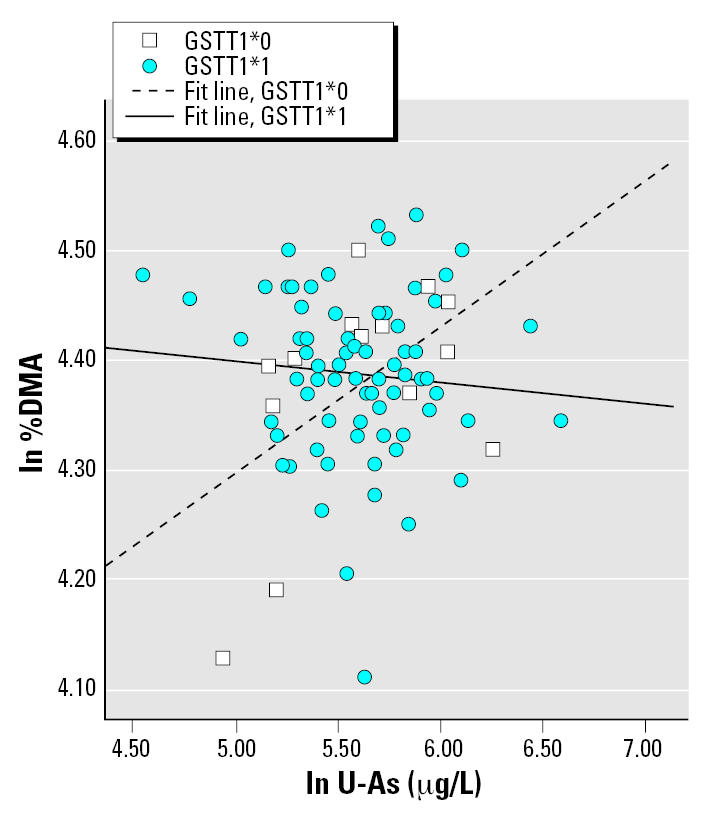
%DMA (ln transformed) as a function of U-As (μg/L) among 2004–2005 women with different *GSTT1* genotypes. Simple regression lines within each genotype group are presented (the regression lines were not derived from the multivariate regression model presented in Table 5; rather, they illustrate the general direction of interaction).

In the multivariate regression models, the influential independent variables were age and U-As for 2004–2005 women (with an interaction term between U-As and genotype) and GW and U-As for pregnant women (interaction term between GW and genotype). The results from the multivariate regression analysis are presented in Table 5; only models yielding *p*-values < 0.1 for the interaction term are shown. No significant interaction was found between *AS3MT* and U-As, but there was still a strong effect of genotype only, with a magnitude similar to the ANOVA tests (data not shown). For the pregnant women, the effects of U-As or GW on As metabolite distribution differed depending on certain genotypes. Carriage of the variant allele of *MTHFR* affected the percentage of all metabolites, as well as the DMA/MMA ratio (%DMA is shown in Figure 2). For *MTR*, there were indications of interactions with U-As among 2004–2005 women (Figure 3) and GW among pregnant women: the heterozygotes were associated with larger β coefficients for %MMA. We observed significant interaction between *GSTM1* and U-As for %MMA (Figure 4) and DMA/MMA, whereas interaction between *GSTT1* and U-As was observed for %MMA, %DMA (Figure 5), and DMA/MMA.

We observed no effect on the MMA/iAs ratio for any genotype studied (data not shown).

## Discussion

This study clearly shows that a significant part of the variation in urinary As metabolite distribution in the Andean people is due to hereditary differences in As metabolism–related genes. We found three strongly linked intronic SNPs in *AS3MT* that significantly affected the As metabolite pattern. Carriers of the variant alleles, especially homozygotes, had lower %MMA and higher %DMA, and consequently, a higher DMA/MMA ratio. For example, variant homozygotes of the SNP35991 had about half the %MMA compared with wild-type homozygotes. Among individuals with more documented indigenous origin, the variant homozygotes had only about one-third of the %MMA compared with wild-type homozygotes. The %iAs was also lower in carriers of variant alleles, although not significantly. This indicates that the effects of the SNPs are strongly associated with the second methylation step in particular. The first methylation step, which is less sensitive than the second methylation step to factors such as sex, excess exposure to As, alcohol, and smoking, may be catalyzed by several different methyltransferases. It may even occur without an enzyme (Zakharyan and Aposhian 1999). Haplotype analysis revealed that the lowest mean %MMA and highest mean %DMA was found among individuals showing homozygosity for the variant allele of all three SNPs. However, there appeared to be no interaction effect between U-As and *AS3MT* genotype.

The three *AS3MT* polymorphisms were also found frequently in another study (Meza et al. 2005) involving Mexican subjects with European and indigenous ancestry (Table 1). That study established a significant association between the possession of at least one variant allele for 12390 and DMA/MMA ratios, but only among children. The authors suggested that the variant has a developmentally regulated effect on As metabolism. However, there was a difference in ethnicity between the groups: The children in the study were of indigenous ancestry to a greater extent, and the adults were more of European ancestry (Meza et al. 2005). In the present study, we genotyped individuals from villages where the people had a more documented Hispanic ancestry. The genotype distribution of the *AS3MT* SNPs in these villiages was significantly different (data not shown), and was similar to the distribution found in those with European ancestry. Our findings from indigenous Andean women strongly indicate that these variants represent a haplotype for indigenous ancestry associated with a specific As-metabolism profile, irrespective of age.

Wood et al. (2006) and Drobna et al. (2004) estimated the functional impact on AS3MT enzyme activity of genetic variants occurring in exons. For intronic polymorphisms, the function on AS3MT activity is still completely unknown, and there are no indications that they are linked to exonic variants within *AS3MT* in the SAC population. Only two individuals from SAC, both pregnant women, were heterozygous carriers of the high-activity variant Met287Thr reported by Wood et al. (2006). These women had rather divergent percentages of MMA: 1.4% and 9.0%.

Pregnancy affects the metabolite pattern by increasing the %DMA in urine with increasing gestational age, probably in concert with the induction of other methylation reactions required for tissue growth and development (Concha et al. 1998b). Therefore, the pregnant women were analyzed separately in our study. We observed fairly similar associations between *AS3MT* genotype and metabolite distribution in this group, but the results were not as strong as for the 2004–2005 group. This may be due to the smaller number of pregnant subjects. However, increased gene expression of AS3MT, possibly also other methyltransferases, during pregnancy are likely to blur the association between the genetic variants of the gene and metabolite pattern.

We also saw effects on As-metabolism for other genotypes. However, these results should be considered as preliminary because they were not as strong as the results for *AS3MT*. In this “explorative” study, multiple testing was performed. Multiple polymorphisms and metabolites were considered, and several multivariate regression models were tested. We are aware of the problems with false-positive findings, which are influenced by the significance level, statistical power, and prior credibility of the association tested (Wacholder et al. 2004). We did not formally adjust the significance level. Sometimes, the statistical power was low because of the small numbers of individuals (e.g., among pregnant women). Problems with false negatives may also be of concern.

Studies on mice and rabbits have shown that chemical inhibition of SAM-dependent methylation reactions result in a marked decrease in the methylation of As to DMA (most mammals do not excrete MMA in the urine), as well as increased tissue concentrations (Marafante and Vahter 1984; Marafante et al. 1985). Our multivariate regression analysis indicated that polymorphisms of the *MTHFR* and *MTR* genes, which are involved in the remethylation of homocysteine to methionine, affected the metabolite pattern among pregnant women. The *MTHFR* 222Val allele has been associated with reduced enzyme activity and higher plasma homocysteine levels (Frosst et al. 1995). As expected, in the multivariate analysis, individuals with one or two variant alleles of *MTHFR* had lower %DMA and higher %iAs and %MMA, compared with the wild type. On the other hand, the interaction terms showed an increase in %DMA (Figure 2) and a decrease in %iAs and %MMA with increasing gestational age, compared with the wild type. There are alternative pathways for the remethylation of homocysteine, mainly via choline/betaine, which may compensate fairly well for loss of the folate/vitamin B12 pathway (Stead et al. 2006) when the body is not under particular stress. Possibly, up-regulation of substituting pathways during pregnancy may balance out lower activity of MTHFR among carriers of the variant allele. For *MTR*, we observed an effect of both the genotype and the interaction term on %MMA for heterozygotes, but in opposite directions. No variant homozygotes were detected among the pregnant women. The variant allele of *MTR* has been found to be a risk factor for cardiovascular disease and for cancer (Klerk et al. 2003; Lin et al. 2004; Zhang et al. 2005). We observed similar but nonsignificant effects of *MTHFR* and *MTR* (Figure 3) polymorphisms on metabolite pattern in the 2004–2005 group. However, these findings for the folate-related genotypes need to be confirmed in a larger study.

Also *GSTM1* (%MMA and DMA/MMA) and *GSTT1* (%MMA, %DMA, and DMA/MMA) modified the metabolite pattern. Marcos et al. (2006) found that GSTM1*0 individuals excreted more MMA, but we did not see this effect in the present study using ANOVA; however, GSTM1*0 carriers had higher fractions of MMA with increasing U-As. Chiou et al. (1997) reported an association between high %iAs and GSTM1*0. We found no clear effects on %iAs for this genotype in 2004–2005 women (*p* = 0.7) or in pregnant women (*p* = 0.4). Unlike Chiou et al. (1997), we found no association between high %DMA and GSTT1*0.

For *GSTO1*, the frequencies of polymorphisms were far too low to affect the distribution of urinary As metabolites among the population. The effect of the novel *GSTO1* 2562 T/C substitution is still to be elucidated. Marnell et al. (2003) showed that two Mexican individuals, both heterozygous for Glu155del and Glu208Lys, displayed significantly higher %iAs in the urine (67–79%). The two individuals in the present study that were heterozygous for Glu155del were also heterozygous for Glu208Lys, but Marnells’ findings of a high %iAs in the urine for this genotype was not verified. Many other factors, such as exposure level, age, and diseases, may influence the metabolism of As (Vahter 2002).

The population from SAC has been exposed to As for thousands of years (Figueroa et al. 1992; Núñez et al. 1991), and we speculate that this has resulted in a positive selection for genotypes that metabolize As in a less toxic way. The frequencies of the polymorphisms in *AS3MT,* associated with increased formation and excretion of DMA in urine, were very high compared with other populations (Meza et al. 2005; Wood et al. 2006). Little is known about the prevalence of As-associated health effects in this population. We did not find a single case of As-induced hyperkeratosis on the palms of the hands among the studied women, although in a previous study (Dulout et al. 1996) we observed an increased frequency of micronuclei and of trisomy in lymphocytes from exposed children and women compared with nonexposed controls. The Andean women also seem to have lower blood/urine ratios than other populations (Concha 2001). Also, As-induced skin lesions have been observed in Atacameño people in northern Chile, although mainly in men (Smith et al. 2000), who are known to be more sensitive to As-induced skin lesions than women (Rahman et al. 2006). Obviously, a more comprehensive investigation is needed on the health consequences of As exposure in SAC.

Identifying polymorphisms, gene–environment interactions, and related effects on As metabolism will provide important information about the mechanisms behind the biotransformation of As and also facilitate understanding individual differences in As metabolism. This will help us to identify susceptible groups and may provide better risk estimates for As. Furthermore, this research has clinical implications in that it may be used for evaluation of the risks associated with the current use of arsenicals as a contemporary pharmaceutical agent.

## Figures and Tables

**Table 1:** Genotype and allele frequencies (%) for all individuals from SAC and previously reported genotype frequencies from other studies.

SNP, Genotype^a^	SAC^b^ [genotype % (allele %)]	Indigenous American ancestry	European/Hispanic ancestry
*AS3MT* 12390	*n* = 112	*n* = 22	*n* = 24
GG/GC/CC (G/C)	6/43/51 (28/72)	NA (58/42)^c^	NA (80/20)^c^
*AS3MT* 14215	*n* = 145	*n* = 22	*n* = 24
CC/CT/TT (C/T)	6/35/59 (28/72)	NA (62/38)^c^	NA (90/10)^c^
*AS3MT* 35991	*n* = 146	*n* = 22	*n* = 24
GG/AG/AA (G/A)	6/36/58 (24/76)	NA (50/50)^c^	NA (52/48)^c^
*GSTM1*	*n* = 139		*n* = 23
GSTM1*1/*0	39/61	NA	72/28^d^
*GSTT1*	*n* = 122		*n* = 23
GSTT1*1/*0	83/17	NA	65/35^d^
*MTR*	*n* = 147		*n* = 23
AA/AG/GG (A/G)	71/28/1 (85/15)	NA	61/30/9 (76/24)^d^
*MTHFR*	*n* = 145		*n* = 23
CC/CT/TT (C/T)	43/43/14 (64/36)	NA	48/43/9 (70/30)^d^

NA, not available.

a Wild-type allele denoted first.

b The number of individuals with genotype information varies due to insufficient amounts of DNA (mainly for *AS3MT* 12390 and *GSTT1*).

c Data from Meza et al. (2005).

d Data from NCI (2006).

**Table 2:** Median metabolite and U-As values.

Group	No. of cases with metabolite data	%iAs (range)	%MMA (range)	%DMA (range)	U-As [μg/L (range)]
Pregnant women^a^	37 of 37	14 (4.5–27)	3.1 (0.1–9.0)	83 (69–92)	233 (111–401)
2004–2005 women^b^	110 of 111	12 (1–48)	7.4 (1.2–18)	80 (47–93)	272 (94–724)

a Data from Concha et al. (1998b); pregnant women from 2004 and 2005 were included.

b Pregnant women were excluded.

**Table 3:** Geometric mean As metabolite values (%), ratios of MMA/iAs and DMA/MMA and U-As for different genotypes for 2004–2005 women.

Polymorphism^a^	%iAs	%MMA	%DMA	MMA/iAs	DMA/MMA	U-As	No.
*AS3MT* SNP35991^b^							
GG	16.7	13.6	68.3	0.82	5.0	285	5
GA	11.8	7.8*	77.3*	0.66	9.9**	280	43
AA	11.0	6.6#	80.4#	0.60	12.2#	261	61
*p*-Value	0.22	< 0.001	< 0.001	0.5	< 0.001	0.6	
*MTR*							
AA	11.2	7.3	79.0	0.65	10.8	270	79
AG	12.0	7.0	79.0	0.58	11.4	272	29
GG	21.7	14.9*	62.2**	0.69	4.2	228	2
*p*-Value	0.20	0.047	0.006	0.7	0.016	0.8	

a Only SNPs with *p*-values < 0.1 are presented.

b All *AS3MT* SNPs gave very similar results; therefore, only SNP35991 is presented.

* *p* < 0.05,

** *p* < 0.01, and

# *p* < 0.001 compared with wild type.

**Table 4:** Geometric mean As metabolite values (%), ratios of MMA/iAs and DMA/MMA and U-As for different genotypes for pregnant women.

Polymorphism^a^	%iAs	%MMA	%DMA	MMA/iAs	DMA/MMA	U-As	No.
*AS3MT* SNP35991^b^							
GG	20.9	4.3	73.7	0.21	16.9	185	4
GA	13.7	2.9	80.6	0.21	27.0	215	9
AA	13.5	2.1	82.2*	0.16	46.9	245	24
*p*-Value	0.15	0.30	0.033	0.7	0.2	0.3	
*GSTT1*							
GSTT1*1	13.3	2.2	81.5	0.18	37.0	230	29
GSTT1*0	18.2	3.5	76.7	0.22	21.6	219	7
*p*-Value	0.092	0.23	0.051	0.8	0.18	0.8	
*MTR*							
AA	13.1	2.3	82.2	0.18	35.9	270	26
AG	16.9	3.0	78.2	0.18	26.3	270	11
*p*-Value	0.084	0.43	0.079	1.0	0.4	1.0	

a Only SNPs with *p*-values < 0.1 are presented.

b All *AS3MT* SNPs gave similar results; therefore, only SNP35991 is presented.

* *p* < 0.05 compared with wild type.

**Table 5:** Multivariate regression modeling of effect modification by some genotypes on the distribution of As metabolites (log-transformed).

	β	SE	*p*-Value
*MTHFR*			
Pregnant women (*n* = 33)^a^
iAs (*R*^2^)^b^		0.33	
Intercept	1.1	0.95	0.27
Genotype 0/1^c^	0.97	0.48	0.052
Genotype0/2^c^	1.2	0.54	0.040*
U-As	0.24	0.17	0.17
GW	0.008	0.014	0.6
Genotype*GW 0/1^c^	−0.032	0.018	0.094
Genotype*GW 0/2^c^	−0.036	0.020	0.081
MMA (*R*^2^)		0.21	
Intercept	0.20	2.4	0.9
Genotype 0/1^c^	2.5	1.2	0.050*
Genotype 0/2^c^	2.1	1.4	0.14
U-As	−0.059	0.44	0.9
GW	0.039	0.036	0.3
Genotype*GW 0/1^c^	−0.102	0.047	0.042*
Genotype*GW 0/2^c^	−0.085	0.051	0.11
DMA (*R*^2^)		0.46	
Intercept	4.7	0.17	< 0.001
Genotype 0/1^c^	−0.30	0.087	0.002**
Genotype 0/2^c^	−0.31	0.099	0.004**
U-As	−0.026	0.031	0.4
GW	−0.004	0.003	0.17
Genotype *GW 0/1^c^	0.010	0.003	0.006**
Genotype*GW 0/2^c^	0.010	0.004	0.010**
DMA/MMA (*R*^2^)		0.24	
Intercept	4.5	2.5	0.086
Genotype0/1^c^	−2.8	1.2	0.033*
Genotype0/2^c^	−2.4	1.4	0.099
U-As	0.033	0.45	0.9
GW	−0.042	0.036	0.25
Genotype*GW 0/1^c^	0.112	0.049	0.029*
Genotype*GW 0/2^c^	0.095	0.052	0.078
*GSTM1*			
2004–2005 women (*n* = 100)
MMA (*R*^2^)		0.18	
Intercept	2.8	0.95	0.004
Genotype^d^	−4.4	1.3	0.001#
U-As	−0.12	0.17	0.5
Genotype*U-As^d^	0.79	0.23	0.001#
Age	−0.005	0.003	0.1
DMA/MMA (*R*^2^)		0.17	
Intercept	1.3	1.1	0.2
Genotype^d^	4.6	1.5	0.001#
U-As	0.15	0.19	0.4
Genotype*U-As^d^	−0.85	0.26	0.002#
Age	0.006	0.003	0.067
*GSTT1*			
2004–2005 women (*n* = 84)			
MMA (*R*^2^)		0.12	
Intercept	−0.31	0.83	0.7
Genotype^d^	3.8	1.8	0.038*
U-As	0.41	0.15	0.006
Genotype*U-As^d^	−0.65	0.32	0.047*
Age	−0.002	0.003	0.5
DMA (*R*^2^)		0.11	
Intercept	4.4	0.16	< 0.001
Genotype^d^	−0.89	0.34	0.011*
U-As	−0.017	0.028	0.5
Genotype*U-As^d^	0.16	0.06	0.011*
Age	0.001	0.001	0.073
DMA/MMA (*R*^2^)		0.12	
Intercept	4.7	0.92	< 0.001
Genotype^d^	−4.7	2.0	0.021*
U-As	−0.42	0.16	0.010
Genotype*U-As^d^	0.81	0.36	0.026*
Age	0.003	0.004	0.4
*MTR*			
2004–2005 women (*n* = 106)^e^			
MMA (*R*^2^)		0.17	
Intercept	1.36	0.75	0.074
Genotype 0/1^c^	−2.82	1.5	0.058
Genotype 0/2^c^	1.5	3.0	0.6
U-As	0.14	0.133	0.3
Genotype*U-As 0/1^c^	0.49	0.26	0.066
Genotype*U-As 0/2^c^	−0.15	0.54	0.8
Age	−0.004	0.003	0.16
Pregnant women (*n* = 33)			
MMA (*R*^2^)		0.19	
Intercept	1.4	2.6	0.6
Genotype^c^	−2.1	1.3	0.11
U-As	0.096	0.46	0.8
GW	−0.048	0.022	0.034
Genotype*GW^c^	0.094	0.048	0.059

For 2004–2005 women, the model contains effect modification by genotype, U-As, and age on the distribution of As metabolites, with an interaction term of U-As*Genotype. For pregnant women, the model contains effect modification by genotype, U-As, and GW on the distribution of As metabolites, with an interaction term of GW*Genotype. AS3MT polymorphisms are not included because no interaction effect was found.

a GW data are missing for four individuals.

b Proportion of the total variance of the metabolite levels (ln transformed); values are unadjusted.

c Individuals with no variant alleles were used as referents and were compared with those with 1 and 2 variant alleles, respectively. In the pregnant women, no individuals with two variant MTR alleles were found.

d GSTM1*1/GSTT1*1 were used as referents compared with GSTM1*0/GSTT1*0.

e U-As data are missing for four 2004–2005 women.

* *p* < 0.05,

** *p* < 0.01, and

# *p* < 0.001 compared with wild type.
